# P71. Adoptive transfer of TCR gene-transduced lymphocytes targeting MAGE-A4 for refractory esophageal cancer

**DOI:** 10.1186/2051-1426-2-S2-P45

**Published:** 2014-03-12

**Authors:** H Shiku, H Ikeda, Y Miyahara, M Ishihara, N Katayama, D Tomura, I Nukaya, J Mineno, K Takesako, S Kageyama

**Affiliations:** 1 Mie University Graduate School of Medicine, Cancer Vaccine and Immuno-Gene Therapy, Tsu Mie, Japan; 2 Mie University Graduate School of Medicine, Hematology and Oncology, Tsu Mie, Japan; 3 Takara Bio Inc., Center for Cell and Gene Therapy, Shiga Mie, Japan

## Background

Engineering the antigen receptor gene in patients' lymphocytes is one promising strategy to create antigen-specific lymphocytes without senescent phenotypes. The strategy provides an opportunity to extend the application of adoptive T cell therapy for cancer patients. However, this concept has not been tested in the epithelial cancer patients.

## Materials and methods

MAGE-A4-specific TCR α and β chains were cloned from a human T cell clone that recognises MAGE-A4_143-151_ peptide in a HLA-A*24:02 restricted manner. This T cell clone did not show any cross reactivity to the peptides with homology to the MAGE-A4_143-151_ epitope. A retroviral vector that encodes these TCR chains without any artificial modification was constructed; the lymphocytes transduced with the retroviral vector killed the MAGE-A4 expressing tumor in vitro and inhibited the tumour growth in the NOG immunodeficient mice.

A phase I clinical trial of TCR gene therapy targeting MAGE-A4 was performed to treat refractory esophageal cancer patients without lympho-depleting pre-conditioning. The trial was designed as a cell-dose escalation consisting of three cohorts, 2x10^8^, 1x10^9^ and 5x10^9^ cells/patient. Vaccines with the cognate peptide were also given following adoptive transfer of lymphocytes on day 14 and day 28.

## Results

The treatment was tolerable with no adverse events associated with transferred cells or any viral toxicity. In all ten patients of the 3 cell-doses, the transferred lymphocytes were detected in their peripheral blood in a dose-dependent manner during the first 14 days. In 4 patients, the infused cells have been persisting more than 5 months after the transfer. The transferred lymphocytes that were harvested from the patients more than 50 days after the transfer were found to sustain the reactivity to the antigen-expressing tumour cells. Three patients showed SD or long tumour free status.

## Conclusions

This approach may extend the availability of adoptive T cell therapy for epithelial cancer patients by providing tumour-reactive and long surviving lymphocytes.

